# Do Training Load Metrics Agree? A Comparison of Session Rate of Perceived Exertion, Physiological and Biomechanical Load in Outdoor Running

**DOI:** 10.1186/s40798-025-00969-9

**Published:** 2026-02-27

**Authors:** Bouke L. Scheltinga, Jaap H. Buurke, Joost N. Kok, Jasper Reenalda

**Affiliations:** Biomedical Signals and Systems, Faculty of Electrical Engineering, Mathematics and Computer Science (EEMCS), University of Twente, Enschede, The Netherlands

**Keywords:** Training load, Running, Cumulative load, Physiological load, Ground reaction force, Inertial measurement unit, Biomechanical load, Rate of perceived exertion

## Abstract

**Background:**

Monitoring training load is an important aspect of optimizing performance and preventing overuse injuries in runners. This is the first study comparing physiological, biomechanical and subjective load between typical outdoor training sessions, contributing to the transfer of methodologies from the gait laboratory to real-world conditions with the final goal of improving athlete monitoring.

**Methods:**

Twelve experienced runners participated in distinct sessions: an endurance run, a submaximal effort, and interval training, which varied in perceived exertion. Using heart rate monitors, inertial measurement units and questionnaires, estimated cumulative load and its correlation with session Rate of Perceived Exertion (sRPE) and physiological load calculated via Training Impulse (TRIMP) were analysed.

**Results:**

sRPE significantly distinguished between session types, while TRIMP and cumulative biomechanical load did not. Furthermore, correlations between the three training load metrics were weak to moderate (sRPE vs. TRIMP: *r* = 0.49; sRPE vs. weighted cumulative load: *r* = 0.25; weighted cumulative load vs. TRIMP: *r* = 0.35), where only sRPE and TRIMP correlated significantly (*p* < 0.05). This suggests that the different measures capture different aspects of load or that the measures could be inadequate to capture load.

**Conclusion:**

Objective physiological and biomechanical metrics alone may not adequately reflect athletes’ perceived exertion when training includes different session types. This highlights the importance of using a multifactorial approach to training load monitoring in running.

## Key Points

- In contrast to TRIMP and biomechanical load, sRPE distinguished between typical running sessions in recreational runners.
- TRIMP and sRPE showed moderate correlation over various training sessions, while cumulative load was not significantly correlated with the other load metrics.

## Background

Running is a popular physical activity known for its significant health benefits, both physically [1] and mentally [2]. A major challenge for runners is the risk of injury. Reported injury rates remain high, and the effectiveness of injury-prevention interventions is limited, despite decades of research [3–6]. While various factors contribute to running injuries, one commonly proposed mechanism is the accumulation of mechanical stress on tissues over time, which may lead to overuse injuries similar to material fatigue [7]. This has led to a growing interest in monitoring training to better understand its role in both performance optimization and injury prevention [8].

Historically, athletes assessed training load using metrics such as total distance covered and time spent running. These external measures provided a simple estimate of training volume but lacked insight into forces and physiological responses within the body [9]. Since the 1980s, heart rate monitors were used as a tool for monitoring physiological training load in running [10], enabling athletes to monitor their heart rate continuously and gain insights into the physiological response of their bodies during exercise. The heart rate monitors provided a way to measure the intensity of exercise objectively and set individualised training zones based on an individual’s heart rate response to exercise [11].

One commonly used HR-based load measure is Training Impulse (TRIMP), which integrates both the intensity and volume of an activity into a single measure [12]. This method allowed for a standardized method to quantify physiological training load and even methods to model performance, offering insights into workload management and performance prediction [13, 14]. This helps athletes and coaches to tailor their training plans to their individual physiological responses to optimise performance and prevent overtraining [15, 16].

Training load is typically divided into physiological load, defined as the stress placed on body’s physiological systems and biomechanical load, defined as physical stresses acting on the body or on anatomical structures within the body [17, 18]. Unlike physiological load, biomechanical load has been more challenging to measure outside of the laboratory [19]. As a result, most biomechanical research has relied on controlled environments, limiting the ability to translate knowledge and methods to real-world conditions [19]. Advances in inertial measurement units (IMUs) allow measuring the biomechanics of runners in the outdoor environment. IMUs make it possible to measure kinematics during a marathon and quantify changes as a result of fatigue [20, 21]. Many studies have focused on quantifying biomechanical parameters using a reduced sensor setup with the ability to use it outdoors. For example, with three inertial measurement units placed at the lower legs and pelvis, ground reaction force (GRF) can be estimated [22, 23]. Recently, IMUs have been utilized to even estimate joint loading [24], marking a step towards monitoring structure specific load.

Most of these studies, however, have been performed on a treadmill, where the environment is controlled and the velocity is constant. Also, kinematic differences are shown between overground and treadmill running [25]. Treadmill running also provokes a higher physical demand than overground running, as both RPE and heart rate are higher with the same running speed [26]. Because of these reasons, it is suggested that future studies should move out of the lab and measure in outdoor conditions [19, 27]. Recently, the repeatability of an algorithm for GRF estimation was found to be good, supporting its potential for application in real-world conditions beyond controlled laboratory settings [28].

To quantify cumulative biomechanical load, mechanical fatigue models to estimate the accumulation of mechanical stress over time could be applied. One approach introduced by Edwards, derived from the Palmgren-Miner rule, sums peak stress raised to the power of a constant b [7]. This force summation is based on the principle that biological tissues experience fatigue damage nonlinearly, where larger forces contribute disproportionately more to total loading, accelerating tissue damage and potentially increasing injury risk.

Previous studies have typically used b = 9, derived from in vitro Achilles tendon experiments [29]. However, this choice remains uncertain when applied to whole-body GRF in running. Kiernan et al. investigated weighted cumulative load using estimated peak GRF longitudinally and concluded that cumulative loading metrics may be a valid approach to assess injury risk [30]. However, the relationship between cumulative force-based load measures and physiological or subjective training load remains poorly understood.

In addition to objective physiological and biomechanical measures, subjective load assessment remains an essential tool for monitoring training intensity [31]. The rating of perceived exertion (RPE), when multiplied with activity duration in minutes, results in session RPE (sRPE). sRPE is commonly used as a subjective indicator of training intensity, related to various team sports [32] and known to correlate with blood lactate thresholds [33].

It is unknown how well different load metrics align with subjective perception of effort across different types of running training. Understanding these relationships is essential for determining whether biomechanical, physiological, and subjective measures provide complementary or redundant insights into training load. To date, no studies have systematically compared these load measures in real-world running conditions.

This study aims to compare multiple training load metrics, physiological (TRIMP), biomechanical (weighted cumulative load), and subjective (sRPE), collected during three typical running sessions in an outdoor setting. Specifically, we address the following two research questions:

1. How do different training load metrics quantify different type of training sessions?
2. What is the relationship between load quantified using biomechanical, physiological, and subjective measures? Answering these questions helps in understanding whether commonly used load measures provide consistent, complementary or conflicting estimates of training load.

By analysing physiological, biomechanical, and subjective load simultaneously, this study provides new insights into how different load metrics respond to training in real-world training situations. These findings will help guide best practices for monitoring training load in runners and inform the future development of field-based load management strategies. Furthermore, the collected dataset will be published, enabling future research to compare load metrics across different studies and validate new approaches to training load monitoring.

## Methods

### Participants

Fourteen participants were recruited for this study. Participants were eligible for participation if, in the six months before inclusion, they [1] ran at least 20 km/week [2], ran at least twice a week and [3] had no major running-related injury. A major running-related injury was defined as an injury that lasted longer than 10 days and caused a runner to shorten or skip runs because of this injury. Participants were recruited from local athletics and triathlon associations. The experimental protocol was conducted according to the principles outlined in the Declaration of Helsinki and was approved by the ethics committee of the University of Twente (application number 230439). All participants signed an informed consent form prior to participation.

The participants were asked to visit the University of Twente outdoors athletics track (400 m, tartan running surface) for three sessions, performed in the following order: an endurance session (END), a 5 km submaximal effort (5 K), and an interval training (INT). These three sessions were preferably planned over a period of 3 weeks. Measurements were rescheduled if participants were not available, feeling ill, or the athletics track and/or the surrounding roads were too slippery because of icy and snowy weather conditions. On average, there were 11 days between two measurements for a single subject.

### General Participant Information

During the first visit, general participant information was collected, such as age, sex, height, running participation, running experience, minimum and maximum heart rate and representative race results from a 5–10 km road event. The participant’s body mass was measured in their running outfits, including sensors. Subjects wore their own shoes, the same pair for every session.

### Running Protocol

**Fig. 1:**
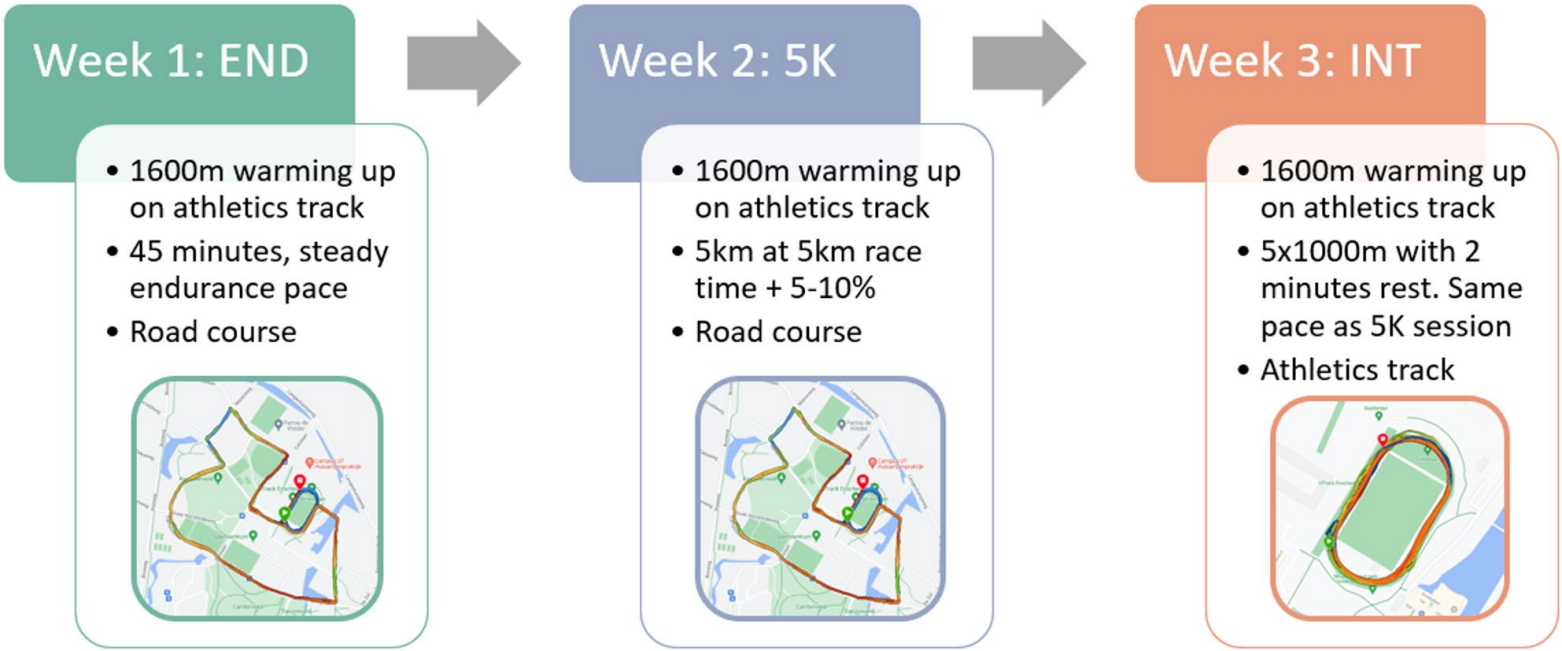
Overview of the protocol and different running sessions

The running protocol is visually shown in Fig. 1. During each visit, the participants started with a warm-up of four laps on the athletics track in a counterclockwise direction. Participants were instructed to run at a constant pace at which they would do a long endurance run. The warm-up data were used in another study to validate the repeatability between the three sessions.

#### Endurance Run

On the first visit, participants continued at the same pace after warm-up, running on a 2.5 km flat road course with less than 5 m of elevation change. Participants were instructed to run laps on the road course at a constant pace and return after 45 min of running, including warm-up on the track.

#### 5 km Submaximal

On the second visit, the participants had to run the road course for two laps at a steady, submaximal pace after warm-up. Participants had up to two minutes’ rest to change clothing and/or drink between the warm-up and the start of the 5 K session. After two laps on the road course, which ended on the athletics track, they kept running on the track until the watch recorded 5.0 km. In practice, participants ran for about 80 m on the athletics track after two laps on the road course.

#### Interval Run

On the third visit, participants completed a 5 × 1000 m interval run on the athletics track with a 2-minute rest. Participants could choose how to spend the rest (walk/jog). They were instructed to run at the same pace as the 5 K session during the intervals. No feedback was given during the session.

#### Pace Calculation

The 5 km submaximal pace was determined by the result of a representative 5 km race event. If only a race result of an event other than 5 km was known, a 5 km race time was estimated using Riegel’s rule:

$${T}_{2}={T}_{1}\left({\frac{{D}_{2}}{{D}_{1}}}\right)^{1.06}$$

where T_1_ and D_1_ represent the time and distance of the known race event and T_2_ and D_2_ the time and distance of the estimated result, in our case, 5 km [34]. The 5 km submaximal trial time was calculated by adding 5–10% to the 5 km race result. The final pace was determined in consultation with the participant. Participants could determine their warm-up and endurance pace. As a rule of thumb, this was at least 1:00 min/km slower than the 5 km submaximal pace. All runs during this research were self-paced.

## Measurement Setup

During each visit, participants were equipped with a Garmin Forerunner 225 GPS watch, (Garmin Ltd., Olathe, Kansas, United States), connected to a heart rate monitor chest strap (HRM-Dual, Garmin Ltd.), participants were allowed to use their own Garmin watch and heart rate monitor if the release date of the watch was after 2018. Additionally, seven Movella DOT IMUs (internal sampling frequency of 800 Hz; accelerometer range of ± 16 g; Movella, Enschede, The Netherlands) were placed at the (proximal) tibias and pelvis. As part of a larger study, more sensors were placed (on the upper legs, sternum and on the wrist underneath the strap of the GPS watch). Before the measurement, the internal clocks of the IMUs were synchronized and a magnetic field mapping was performed to calibrate the magnetometer, both according to the manufacturer’s instructions [35]. The IMUs were fixed using double-sided tape and covered with a piece of Fixomull stretch tape. The GPS watch had locked onto a satellite signal before the start of the run. Watch and heart rate data were collected at 1 Hz, IMU data at 120 Hz. All data were recorded locally on sensors.

All measurements started with a static trial, where the participants stood in a neutral pose (N-pose) for 10 s. To achieve temporal synchronization between the GPS-watch data and the IMUs, the participants jumped prior to running and started the watch at the moment of touchdown.

Directly after running, participants filled out their RPE, using a visual version of the Borg CR10 scale, with a description per score. Additionally, RPE values were asked for specifically physiological and biomechanical load, but these scores were not considered in this manuscript.

### Data Processing

Data from the Garmin watch were exported as .TCX files using the Garmin Connect website [36]. The acceleration in the sensor frame along the axis aligned with the tibia was inspected to find the acceleration peak caused by the jump prior to running. This peak was used for the temporal synchronization between the GPS watch and IMUs.

To assess adherence to the running protocol, per subject, the sum of the five times one kilometer interval time is compared to the 5 km time of the 5 K session and to the quickest 5 km within the END session. Also the average heart rate, total distance and moving time (total time with speed > 1 m/s) were compared between the sessions.

The previously developed model [37] based on Newton’s second law was used to estimate the biomechanical load, represented by the vertical GRF from IMU data from the pelvis and both tibias. The vertical GRF was estimated as:

$$\:GRF=\left({m}_{b}\times\:g\right)+{\sum\:}_{i=1}^{3}{m}_{b}\times\:W{F}_{i}\times\:\left({a}_{z,\:i}\right),$$

with body mass *m*_*b*_, gravitational acceleration *g*, sensor *i*, weight factor *WF*_*i*_ and acceleration in the vertical direction of the global coordinate system of sensor *i*, $\:{a}_{z,\:i}$. The weight factor corresponding to the pelvis data was set to 0.550. The weight factor corresponding to the tibias was set to 0.225. Acceleration in the global coordinate frame was computed internally on the sensor using manufacturer-provided algorithms, and the resulting processed data were output directly by the device. The vertical acceleration was filtered for the pelvis using a 2nd order bidirectional Butterworth filter with a cut-off frequency of 5.97 Hz. The vertical acceleration for the tibias was filtered using a 1 st order bidirectional Butterworth filter with a cut-off frequency of 8.74 Hz. The weight factors and cut-off frequencies were taken from [37], where parameters to estimate GRF were optimized to reduce the error between modelled and measured GRF. Forces lower than 20 N were set to 0 N. The original study demonstrates an RMSE of 0.18 times body weight and peak error of 3.9%, validated on 10, 12 and 14 km/h on stride frequencies ranging from − 10% till + 10% of preferred stride frequencies of the participants. The estimated forces were normalized to body weight (BW). A previous study shows good repeatability (intraclass correlation coefficient of 0.86) of this GRF estimation model [28].

The weighted cumulative load was obtained for running steps only, by first detecting peaks in the GRF profile higher than 1.8 times body weight (BW), with a minimum distance of 0.28 s between the peaks. Weighted cumulative load was calculated as the sum of the 9th powers of the detected GRF peaks [30].

The physiological training impulse was calculated as TRIMP based on the relationship between blood lactate levels and heart rate [38]. TRIMP was calculated as

$$\begin{aligned}\mathrm{TRIMP} & =D\times\:{\Delta\:}HR\, \mathrm{-ratio}\times\:Y, \\ & \:\mathrm{with}{\Delta\:}HR \,\mathrm{-ratio}=\frac{H{R}_{\mathrm{ex}}-H{R}_{\mathrm{rest}}}{H{R}_{\mathrm{max}}-H{R}_{\mathrm{rest}}}\end{aligned},$$

$$\:Y=0.64{e}^{1.92\:*\:{\Delta\:}HR \mathrm{-ratio}}\hspace{1em}\text{(for males),} \,\, \mathrm{and}$$

$$\:Y=0.86{e}^{1.67\:*\:{\Delta\:}HR\mathrm{-ratio}}\hspace{1em}\text{(for females)}.$$

With D the duration of the activity in minutes, normalized heart rate intensity $\:{\Delta\:}HR\mathrm{-ratio}$ calculated using exercise heart rate HR_ex_, resting heart rate HR_rest_ and maximum heart rate HR_max_. Y is an intensity factor that accounts for the metabolic demand of training, based on the exponential rise of blood lactate levels, defined specifically for male and female athletes [38, 39]. For the calculation of TRIMP, only data were included when the participant was moving, defined as a speed greater than 0.5 m/s. For one participant the maximum heart rate was unknown and 220 – age was used instead. The same duration (total moving time) as used in the TRIMP calculation (in minutes), was used to multiply the RPE-score to obtain sRPE.

### Statistical Analysis

A Friedman test was used to assess differences in training load measures across the three sessions for each load metric. When a significant difference was found, Wilcoxon signed-rank tests were used post hoc to identify pairwise differences. Non-parametric tests were chosen as these are more suitable with a small sample size and do not require normally distributed data. Repeated-measures correlation coefficients and significance were calculated to assess within-subject relationships between sRPE, TRIMP and weighted cumulative load. This approach accounts for within-subject variability while retaining the variability introduced by different training sessions, which was necessary to examine relationships across a range of training loads. All tests used a significance level of alpha < 0.05.

## Results

Fourteen participants started the trial but data from twelve participants (7 male, 5 female, 27.9 ± 7.9 years old, 1.83 ± 0.12 m, 72.1 ± 9.4 kg) were included in this study. Two participants only joined the first visit, as they were injured before the second visit and were therefore excluded from the results. The population was well trained, with an average of 10.1 ± 7.0 years of running experience and 39 ± 19 km/week.

**Fig. 2:**
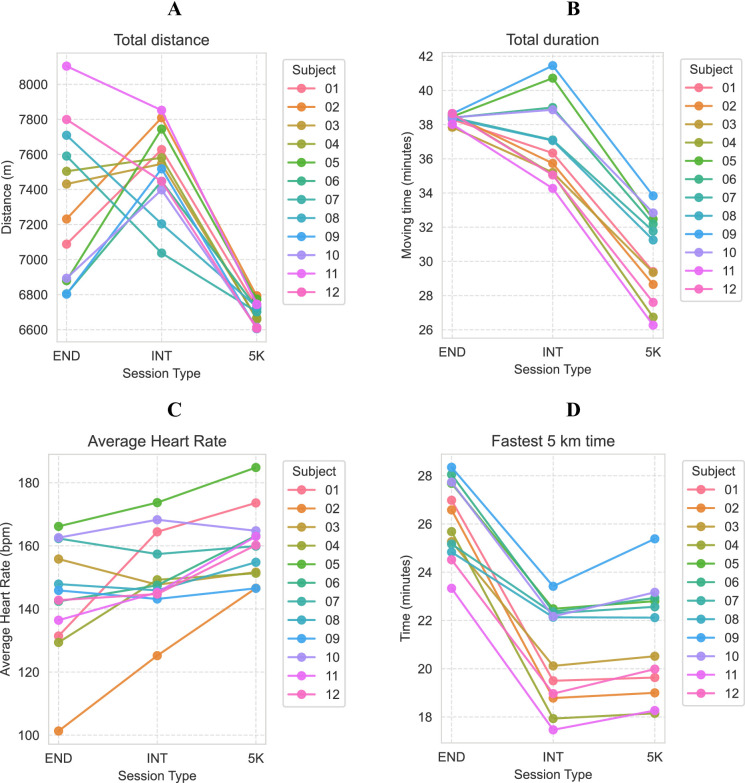
Variation in total distance (A), total duration (B), average heart rate (C) and fastest 5 km time (D) per subject for the endurance (END), interval (INT) and submaximal (5 K) session. Five km time indicates the fastest five km within the END session, the completion time of the five km effort in the 5 K session and the sum of the five kilometer intervals in the INT session. The average for heart rate was taken over data where the moving velocity was > 0.5 m/s

Participants covered most distance in the interval session, followed by the endurance run (7.5 vs., 7.3 km, Table 1; Fig. 2). The 5 K had the shortest duration and covered distance (6.7 km), though, with a higher average heart rate compared to the INT and END session (160 vs. 151 vs. 143 bpm, Table 1) over the complete activity. Adherence to the instructions regarding velocity was analysed by comparing the quickest 5 km within the END session, with the time of the 5 K session and the sum of the five kilometer intervals of the INT session (Fig. 2). All participants ran faster during the INT session compared to the 5 K session. On average, the participants ran 298 s (23%) slower during the END session (26:10) compared to the 5 K session (21:12). The sum of the kilometer interval times from the INT session resulted in the quickest 5 km with 20:38.

**Fig. 3:**
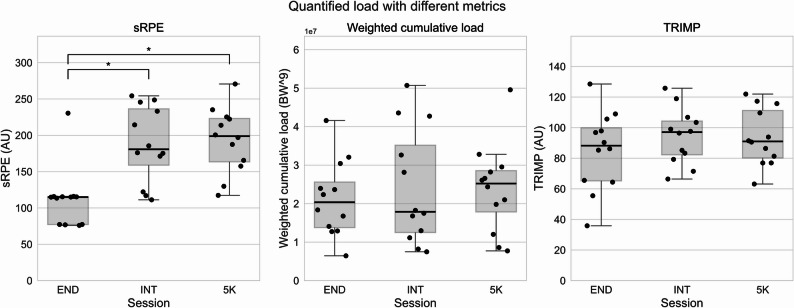
Boxplot of the session rate of perceived exertion (sRPE) based on questions asked directly after each running session, weighted cumulative load based on peak GRF and Training Impulse (TRIMP) based on heart rate. The interval (INT) and submaximal (5 K) differed significantly from the endurance (END) session for sRPE. No significant differences were found between the sessions for weighted cumulative load and TRIMP. The black dots represent individual responses. The box shows the quartiles of the dataset, the black line the mean, and the whiskers extend to 1.5 times the standard deviation. The horizontal bars with “*” indicate statistically significant differences (*p* < 0.05)

The three sessions were experienced differently according to the sRPE (Fig. 3; Table 1). The 5 K session had the highest average sRPE (193.6 ± 44.7) followed by the INT (187.9 ± 52.2). The END session had an average sRPE of 112.0 ± 41.6, with all but one participant reporting an RPE-score of 2 or 3 for the entire activity. The Friedman test indicated differences between the sRPE for sessions, the post hoc analysis revealed significant differences between the END and the other sessions (Table 2). There were no significant differences between the sessions quantified using TRIMP and weighted cumulative load (Table 2).

**Fig. 4:**
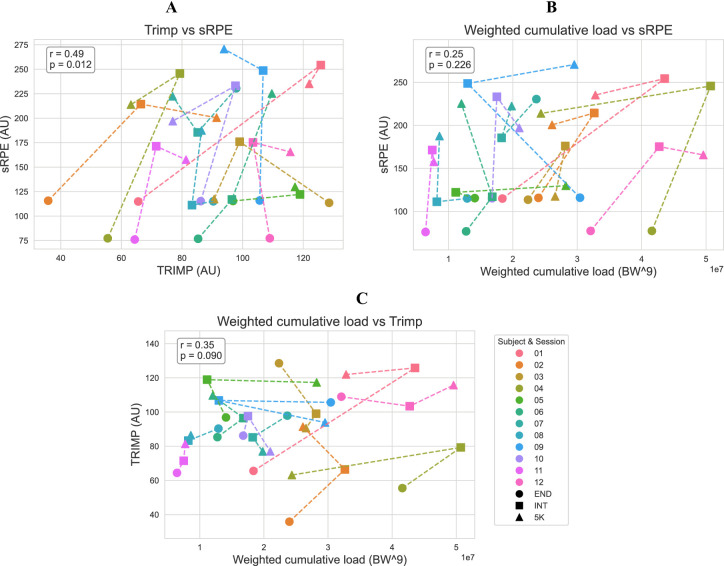
Relation between TRIMP and sRPE (A), weighted cumulative load and sRPE (B), and weighted cumulative load and TRIMP (C). Each sample represents a trial of a single subject. Different colours represent subjects and the shape represents the session type. Lines are connected in the order endurance (END), interval (INT) and 5 km sub maximum run (5 K). Repeated measures correlation coefficient (r) and p-values are added per plot in the left top corner. Only TRIMP and sRPE are significantly correlated (r = 0.49, p-value < 0.05). Session rate of perceived exertion (sRPE), training impulse (TRIMP) based on heart rate and weighted cumulative load based on peak ground reaction force.

The relationship between the three load metrics were visualized (Fig. 4) and quantified using repeated-measures correlation coefficients. A moderate and significant within-subject correlation was found between sRPE and TRIMP (*r* = 0.49, *p* < 0.05), while correlations between sRPE and weighted cumulative load, and between TRIMP and weighted cumulative load, were not statistically significant (*p* > 0.05, Fig. 4).

**Table 1:** Averages ± standard deviations of training metrics over the endurance (END), interval (INT) and submaximal (5 K) sessions

Session	END	INT	5 K
Total distance (km)	7.3 ± 0.4	7.5 ± 0.2	6.7 ± 0.1
Moving time (mm: ss)	38:23 ± 00:14	37:09 ± 02:21	30:11 ± 02:32
Average speed (m/s)	3.2 ± 0.2	3.4 ± 0.2	3.7 ± 0.3
5 km time (mm: ss)	26:10 ± 01:36	20:38 ± 02:03	21:12 ± 02:16
Average heart rate (bpm)	143.7 ± 18.0	151.0 ± 13.1	160.0 ± 11.2
Average cadence (steps/min)	83.9 ± 3.5	80.1 ± 2.4	86.5 ± 3.8
RPE (1–10)	2.9 ± 1.1	5.1 ± 1.4	6.4 ± 1.4
sRPE (AU)	112.0 ± 41.6	187.9 ± 52.2	193.6 ± 44.7
TRIMP (AU)	85.1 ± 25.8	94.5 ± 18.1	93.8 ± 18.6
Weighted cumulative load (BW^9)	(2.13 ± 0.99) × 10⁷	(2.42 ± 1.50) × 10⁷	(2.39 ± 1.15) × 10⁷

Metrics include total distance, duration, speed, heart rate, cadence, perceived exertion (RPE), session RPE (sRPE), physiological load (TRIMP), and Biomechanical load (weighted cumulative load). Five Km time indicates the fastest five Km within the END session, the completion time of the five Km effort in the 5 K session and the sum of the five Kilometer intervals in the INT session. The averages are taken over data where the moving velocity was > 0.5 m/s

**Table 2:** Statistical tests between endurance (END), interval (INT) and submaximal (5 K) session for the session rate of perceived exertion (sRPE), heart rate based training impulse (TRIMP) and peak ground reaction force based on weighted cumulative load

Tested parameter	Test type	Test statistic		*p*-value
		χ^2^	Sum of ranks	
sRPE	Friedman test	10.17		0.006*
	Wilcoxon END vs. INT		5	0.005*
	Wilcoxon END vs. 5 K		2	0.001*
	Wilcoxon INT vs. 5 K		39	1.000
TRIMP	Friedman test	1.50		0.472
Weighted cumulative load	Friedman test	1.17		0.558

If the Friedman test indicated a significant difference within the three groups, a Wilcoxon post hoc analysis was performed. Significant differences (*p* < 0.05) are indicated with “*”. Test statistics are shown as χ^2^ for Friedman and the smallest between the sum of positive and negative ranks for the Wilcoxon test

## Discussion

This study aimed to evaluate how different types of training sessions, endurance, interval, and submaximal, affect training load when measured subjectively (sRPE), physiologically (TRIMP), and biomechanically (weighted cumulative load) in an outdoor running environment. While subjective ratings of perceived exertion differed significantly between the endurance session and other sessions, neither the physiological nor biomechanical load measures reflected significant differences (Fig. 3).

The objective measures of physiological load (TRIMP) showed moderate and statistically significant correlations with subjective ratings of perceived exertion (*r* = 0.49, *p* < 0.05, Fig. 4), while the other metrics did not correlate significantly. The absence of a strong relationship between objective and subjective load measures in this study could be attributed to three factors.

1. **Athletes may not accurately estimate the true load of their activities**.
  This explanation aligns with findings from Kiernan et al. (2018), where runners who eventually sustained an injury had significantly higher cumulative biomechanical load compared to non-injured runners, even though there were no significant differences in their reported pain or fatigue [30]. This suggests that athletes may not always be aware of elevated mechanical strain, particularly when it accumulates gradually over time without immediate discomfort. On the other hand, RPE has been shown to correlate with blood lactate levels during running [33], as well as with percentage of maximum heart rate and maximum oxygen uptake during both cycling and running [40], indicating that individuals’ perceived exertion reflects physiological effort during incremental exercise.
  Within our study, all except one participant scored a 2 or a 3 on the 10 point Borg scale after the END session, corresponding to respectively ‘easy’ or ‘moderate’. Although these values seem to be low, it should be noted that our participant population was well trained. The ratios between average heart rate reserve during the END session and RPE score were comparable to the literature [41], but we could not rule out order effects, as the END session was always performed first.
2. **The used measures used to quantify training load may not be sufficiently adequate.**
  While TRIMP and cumulative load have been previously used to quantify physiological and biomechanical load, respectively, each has limitations. TRIMP is based on heart rate and assumes a consistent relationship between heart rate and blood lactate concentration. However, this relationship varies between individuals and across different types of training [39, 42], with the result that sRPE is a better indicator of blood lactate than TRIMP [42]. This limitation may be addressed by adapting parameters within the calculation of TRIMP to an individual, based on laboratory testing. It was demonstrated that such individualized TRIMP method is more suitable for tracking fitness and performance compared to group based TRIMP [43]. Therefore, this individualized TRIMP probably reflects physiological load better than the group based TRIMP. Additionally, in this study, TRIMP has been calculated using self-reported resting and maximum heart rate, which may have introduced variability in the HR-based load estimates. However, this approach also reflects how HR-based methods are often applied in practice, thereby increasing the ecological validity of our findings.
  Heart rate during exercise was always measured with chest strap heart rate monitors. While the specific models varied per participants, and were not validated against clinical-grade ECG, chest strap devices are generally considered highly accurate [44–46].
  Weighted cumulative load in this study was calculated by summing peak ground reaction forces raised to the ninth power, based on mechanical fatigue theory [7]. This exponent reflects the idea that higher forces disproportionately increase tissue damage, although applying a single exponent to all individuals and tissue types may oversimplify physiological variability. Additionally, this method considers only the peak force during each step, thereby ignoring other important contributors to mechanical load, such as the duration of force application, the rate of loading, and the overall loading pattern throughout the stance phase.
  GRF, used to calculated biomechanical load, is considered as a measure of overall whole-body biomechanical load [47]. However, it does not reflect internal forces due to the high contribution of muscle forces on the internal load [48, 49]. It has been argued that structure specific loads should be used as an input for cumulative load, as structural damage is a direct result of these forces, and that assumptions about internal load based on GRF should be avoided [50]. However, currently there are no methods to estimate internal load reliably in an outdoor environment.
  Finally, weighted cumulative load is highly sensitive; even small changes in estimated force (e.g., due to sensor noise, measurement errors) can be magnified due to the use of an exponential function, potentially leading to large differences in estimated load. This places a high demand on the reliability of the GRF estimation method. Although the method used in this study has demonstrated good repeatability and a peak error of approximately 3.6% [28, 37], any residual error may still be magnified through the power function, particularly at higher force magnitudes. Moreover, the GRF estimation model used in this study was trained on heel-strike runners, which may reduce its accuracy for participants with a midfoot or forefoot strike pattern, potentially influencing the cumulative load outcome in those individuals.
3. **Physiological load, biomechanical load, and perceived exertion might be fundamentally distinct constructs and therefore only weakly related**.
  These three aspects (physiological load, biomechanical load, and perceived exertion) of training load reflect different physiological systems and subjective experiences. For example, a high-speed interval session may involve high mechanical loading (high peak forces) but allows for sufficient recovery to limit cardiovascular strain, resulting in low TRIMP but high cumulative load.
  Perceived exertion adds another layer, shaped by both physical strain and psychological context, such as motivation, sleep, environmental conditions, and prior training [51]. This complexity may explain the moderate and non-significant correlations observed between the objective and subjective measures in this study, supporting the idea that these metrics should be viewed as complementary rather than interchangeable [52]. By capturing the athlete’s subjective response to training, RPE may provide additional insight into perceived difficulty or fatigue that is not apparent in physiological or biomechanical data alone.
  Additionally, differences in training background could further contribute to variability in subjective responses. For instance, an athlete unfamiliar with interval training may perceive the INT session as more demanding than someone who regularly includes such sessions in their training, even if the estimated mechanical and physiological loads are similar. This inter-individual variability highlights the importance of context when interpreting subjective load metrics like sRPE. Where we find a moderate (*r* = 0.49) correlation, previous studies have reported moderate to strong correlations between TRIMP and sRPE in (team) sports [32]. One possible explanation for this discrepancy lies in the design of the current study, where three distinctly different training types (endurance, submaximal, and interval) were chosen to reflect typical variations in recreational running. These session types differ not only in intensity, but also in pace and duration, all of which may influence the perception of effort differently than the physiological response captured by TRIMP. Moreover, it is important to note that TRIMP may not adequately capture physiological fluctuations happening during interval training because of the fluctuations in heart rate [53].

Our findings align with recent work on the user perspective, highlighting that some runners have not much trust in wearables [54]. Runners often prioritize how training feels over what device-generated numbers say and may distrust derived metrics [54]. As subjective monitoring has been shown to provide complementary insight compared with objective metrics [55], future systems should not only communicate objective load data but also include subjective load, which may help to increase users’ trust in wearable technology.

While the intention was to measure the full 45-minute END session using the IMU, corresponding to the memory capacity, the IMUs actually stopped measuring at approximately 90% of this capacity. As a result, the average recorded moving time for the END session was 38:23 min. In practice, participants did complete the full 45-minute session and provided their RPE scores afterward. However, to ensure a fair comparison across load metrics within this session, only the data recorded up to the IMU cut-off point were used. Thus, the final minutes of the session were excluded from the TRIMP and sRPE calculations. This approach may have led to an underestimation of the true sRPE for the END session, but was chosen to maintain consistency across load metrics by aligning all calculations with the available IMU data. For the INT session, a newer version of the sensor with identical sensing hardware and software, but with an increased memory capacity was used to ensure full recording of the protocol.

As measurements were performed during the winter, temperatures between − 10 and 10 °C were seen. With such a temperature difference, it is known that shoe material properties change [56, 57] and alter the shock attenuation of the shoe [58]. This temperature-induced change in shoe properties can influence running kinematics [59]. Besides the effect of the weather on the shoes, the properties of the track are also altered by humidity and temperature [60]. Additionally, on certain measurement days, the track was wet due to rain, decreasing the friction between the shoe and the track, potentially resulting in different running biomechanics. These meteorological factors can influence running biomechanics and, thus, the cumulative load on the athlete.

Moreover, temperature is known to influence the physiological response to exercise. For example, exercise performed in colder temperatures tends to show a lower heart rate compared to the same exercise in a higher temperature [61] affecting the calculation of TRIMP. Additionally, temperature also influences perceived exertion, where exercise in heat is perceived to be harder [62]. Although the impact of temperature is probably small, it can impact both biomechanical and physiological loads.

Validation of methods to estimate physiological load or biomechanical load is very challenging as there is no gold standard. Biomarkers can be used to quantify the physiological response after exercise. For example, insights into inflammatory response or muscle damage after exercise can be obtained using various biomarkers [63, 64]. Future studies incorporating biomarkers for tissue damage could help to validate models for the estimation of biomechanical load [64]. Also, imaging techniques could facilitate the validation of cartilage damage, indicating biomechanical overload. Magnetic resonance imaging (MRI) is capable to quantify changes in the knee and hip joint cartilage after a marathon [65] and knee cartilage response after a walking intervention [66]. A valuable addition for future research is to find relationships between measures of biomechanical load, biomarkers and imaging-based quantifications of load, after a running protocol.

Overuse injuries in running are caused by complex, non-linear, interactions between various factors [67, 68]. Training load is a multi-variate concept and can be divided into physiological, biomechanical, and psychological aspects [52]. This means that it is unlikely to capture stress imposed on an athlete in a single metric that represents all aspects [52]. To increase understanding of the development of running injuries, runners need to be monitored longitudinally using a variety of metrics, representing physiological and biomechanical load as well as subjective measures.

This study shows that, while biomechanical and physiological load metrics may be useful, they fail to reflect perceived exertion across varied training types. This supports the use of a multifactorial approach to in field-based monitoring of training load.

## Conclusions

This study increases our understanding of athlete monitoring in a real-world environment by comparing the weighted cumulative load with physiological load and sRPE during outdoor running. Weighted cumulative load was determined as the sum of the 9th powers of the detected GRF peaks. Both physiological load, as quantified using TRIMP and weighted cumulative load, did not quantify differences between the sessions, though, the 5 K and INT sessions were perceived differently compared to the END sessions, as quantified with sRPE. Additionally, of the tested metrics, only sRPE and TRIMP showed a statistically significant correlation (*r* = 0.49). These findings reinforce the importance of using a multifactorial approach in training load monitoring, one that included the complementarity of subjective, physiological, and biomechanical inputs, rather than expecting them to align perfectly.

## Data Availability

Data and code to reproduce the results of this study are publicly available on the 4TU data repository: 10.4121/efa64223-ba51-48fa-91be-53f0e48460b4. The data analyses can be reproduced by running Python in Docker to ensure that the analysis environment is consistent and reproducible, eliminating issues related to dependency conflicts or system configuration discrepancies.

## Abbreviations

5K: 5 km submaximal effort
BW: Body weight
END: Endurance run
GRF: Ground reaction force
IMU: Inertial measurement unit
INT: Interval training
MRI: Magnetic resonance imaging
RMSE: Root mean squared error
RPE: Rate of perceived exertion
sRPE: Session rate of perceived exertion
TRIMP: Training impulse
